# UMIc: A Preprocessing Method for UMI Deduplication and Reads Correction

**DOI:** 10.3389/fgene.2021.660366

**Published:** 2021-05-28

**Authors:** Maria Tsagiopoulou, Maria Christina Maniou, Nikolaos Pechlivanis, Anastasis Togkousidis, Michaela Kotrová, Tobias Hutzenlaub, Ilias Kappas, Anastasia Chatzidimitriou, Fotis Psomopoulos

**Affiliations:** ^1^Institute of Applied Biosciences, Centre for Research and Technology Hellas, Thessaloniki, Greece; ^2^Department of Genetics, Development and Molecular Biology, School of Biology, Aristotle University of Thessaloniki, Thessaloniki, Greece; ^3^Unit for Hematological Diagnostics, Department of Internal Medicine II, University Medical Center Schleswig-Holstein, Kiel, Germany; ^4^Laboratory for MEMS Applications, IMTEK-Department of Microsystems Engineering, University of Freiburg, Freiburg, Germany; ^5^Hahn-Schickard, Freiburg, Germany

**Keywords:** unique molecular identifiers, molecular barcodes, error correction, next-generation sequencing, bioinformatics

## Abstract

A recent refinement in high-throughput sequencing involves the incorporation of unique molecular identifiers (UMIs), which are random oligonucleotide barcodes, on the library preparation steps. A UMI adds a unique identity to different DNA/RNA input molecules through polymerase chain reaction (PCR) amplification, thus reducing bias of this step. Here, we propose an alignment free framework serving as a preprocessing step of fastq files, called UMIc, for deduplication and correction of reads building consensus sequences from each UMI. Our approach takes into account the frequency and the Phred quality of nucleotides and the distances between the UMIs and the actual sequences. We have tested the tool using different scenarios of UMI-tagged library data, having in mind the aspect of a wide application. UMIc is an open-source tool implemented in R and is freely available from https://github.com/BiodataAnalysisGroup/UMIc.

## Introduction

The introduction of next-generation sequencing (NGS) has revolutionized genomic research and has impacted tremendously clinical applications (Lander et al., 2001; Shen et al., 2015). Through the NGS technologies, researchers are able to study whole genomes (whole-genome sequencing) or smaller regions (exome sequencing), with an unparalleled depth and sensitivity compared to Sanger sequencing (Shen et al., 2015). However, detection of variants with low frequency (below ∼1–3%) still remains a difficult task because of background noise (Fox et al., 2014).

In clinical applications, the detection of true mutants in low-frequency alleles or rare subclones that may contribute to the disease at an early stage remains a big challenge for cancer studies. This is mainly due to the NGS library preparation process, which includes multiple rounds of polymerase chain reaction (PCR) amplification, introducing PCR duplicates and artifacts in the output sequence. This limitation was overcome by the use of unique molecular identifiers (UMIs), facilitating detection and removal of PCR duplicates. Sample preparation involves the introduction of a UMI to each target molecule before PCR amplification. A UMI is a short sequence (usually 8–16 nucleotides, but this can vary depending on the study) that is specific to a molecule and is generated by permutations of a string of randomized nucleotides (Kivioja et al., 2011; Islam et al., 2014).

This method allows monitoring of each target molecule and, consequently, helps reduce PCR amplification bias and increase the accurate quantification and subsequent comparison of targets. UMIs can be used in different NGS methods (Kinde et al., 2011; Salk et al., 2018; Saunders et al., 2020) in a variety of approaches. The most common process to analyze these data is by aligning the sequences to a reference genome or transcriptome with the UMI tag attached to the header. Then, the reads with the same alignment coordinates and UMIs are deduplicated [e.g., UMI-tools (Smith et al., 2017), Picard, zUMIs (Parekh et al., 2018), gencore (Chen et al., 2019), Je (Girardot et al., 2016), etc.]. More recently, tools that skip the alignment step have been developed with a gain in speed on larger datasets [e.g., Calib (Orabi et al., 2019)]. In either scenario, the methods for grouping the reads by their UMIs are similar. The typical process keeps the read that has the highest UMI frequency and the highest quality score (Liu, 2019). Also, many of these tools are not considering the errors introduced to the UMI sequencing and are not be able to estimate the number of true UMIs.

From a different perspective, we have developed a method in which the extraction of the consensus read is performed at the nucleotide level, taking into account the frequency of the bases and their mean quality. We propose an R-based framework, called UMIc, which is a preprocessing step of the raw fastq files based on an alignment-free method. The tool takes as input a fastq file and generates a new fastq file in which each read represents the consensus sequence of a group of unique UMIs. The method for the grouping of reads combines correction of the UMIs and the actual sequence calculating distances between the UMIs and the sequences, respectively. Our approach was tested on empirical UMI-tagged library data from Stahlberg et al. (2017) and Zilionis et al. (2017).

## Materials and Methods

### Overview

Briefly, the workflow of UMIc consists of three main steps:

1. Initial read correction of the sequences with the same UMI, using the previously described method.
2. UMI merging, taking into account both the distance of the UMIs and the distance of the sequences, generated by the first step.
3. Final read correction of the sequences that belong to the same group of merged UMIs, as created by the second step.

Project link: https://github.com/BiodataAnalysisGroup/UMIc

Operating system: Windows

Programming language: R

License: MIT

### Workflow

**FIGURE 1:**
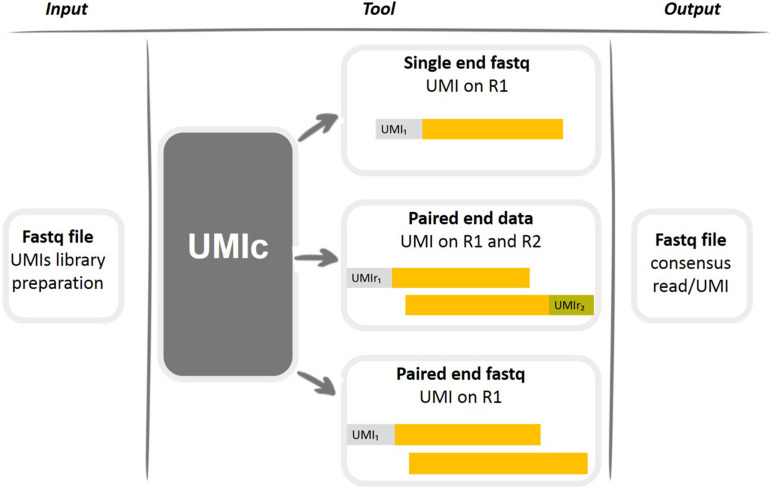
Graphical representation of working cases of the UMIc tool.

UMIc contains one script (*UMIsProject.R*) with another two scripts of dependency including the required functions (*casesWorkflows.R*, *functions.R*). The UMIs have to be attached to the start of the read, and the length depends on the user’s scenarios (user’s option). UMIc can be implemented on three different kinds of libraries (Figure 1): (i) single-read libraries: UMI on R1 (Read1), (ii) paired-end libraries: UMI on R1, and (iii) paired-end libraries: UMI on R1 and R2 (Read2).

The input data must be provided in fastq files, and it is assumed that the UMI is placed at the beginning of each sequence and contains a UMI-tagged library. The output data are stored also in fastq files, with the same name as the input files including the corrected reads (i.e., consensus sequences resulting from the same UMI). The first phase includes the automated process of reading fastq files at a working directory. The library preparation step of the input files must be generated using the same protocol and fulfill the same input parameters described. Then, the workflow consists of the selected criteria by users relating to their analysis (Table 1).

**TABLE 1:** Selected criteria being input from the users.

Option	Type	Details
pairedData	T or F	Boolean variable that indicates whether data are paired T or single F
UMIlocation	R1 or R1 and R2	Variable that indicates whether UMI is located only in R1 or R1 and R2
UMIlength	Numeric	The length of the UMI sequence
sequenceLength	Numeric	The length of the read sequence
countsCutoff	Numeric	Min read counts per UMI, for initial data cleaning
UMIdistance	Numeric	Max UMI distance for UMI merging
sequenceDistance	Numeric	Max sequence distance for UMI merging with the associated reads

### Read Correction Method

We developed a method for the correction of (i) the UMI and (ii) the downstream sequence at the nucleotide level, taking into account the frequency of each base and their mean quality. In more detail, this process can be outlined as follows:

1. Calculation of the frequency and the mean quality of each base.
2. Setting a criterion, defined by the mean of the two previously calculated values of each base.
3. Selection of the base with the maximum value criterion.
4. In case of a draw between bases, selection of the base with the maximum quality value.
5. Setting the new quality as the selected base’s mean quality, calculated in step 1.

These steps result in the generation of a consensus sequence of the initial UMI+read.

### UMI Merging Method

**FIGURE 2:**
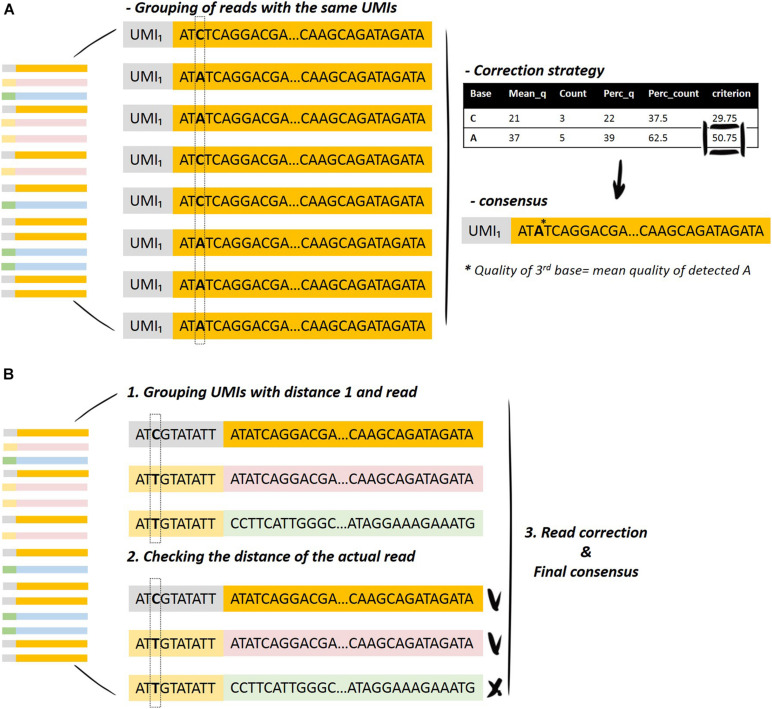
Graphical representation of the UMI merging method. **(A)** Grouping of the reads with the same UMI and selection of the bases for the generation of the consensus sequence. **(B)** Grouping of the UMIs with specific distances and inspection of the distance of the actual read in order to merge the reads resulting from the same UMI.

The process starts with the data-cleaning module. The UMIs that fulfill the condition of minimum reads per UMI are selected for the downstream analysis (the user can set this minimum by changing the parameter countsCutoff). Then, the UMIs are grouped according to specific criteria, and this serves to initiate the deduplication of the reads, keeping the initial sequences resulting from the same UMI. The main idea is described in Figure 2 and in the following steps:

• Finding the UMI with the maximum number of reads.
• Calculation of the distances (Hamming distances) between the UMIs with the maximum number of reads to all the other UMIs, and the same is performed for the corresponding sequence to all the other sequences.
• Finding the reads that fulfill the distance criteria (user selection) and grouping on the UMI with the maximum number of reads.
• Read correction step, as previously described in *Read Correction Method.*
• Removal of the associated reads from the list and continuation of the process with the second UMI with the maximum number of reads.

### Output Files

The output files are stored in a new folder of the working directory named by the user. The output data are stored in fastq files with the same root name as the input files with the addition of the suffix “*_corrected*.” The files contain the corrected sequences (without the UMI) and the corresponding quality resulting from the mean of the selected base.

**FIGURE 3:**
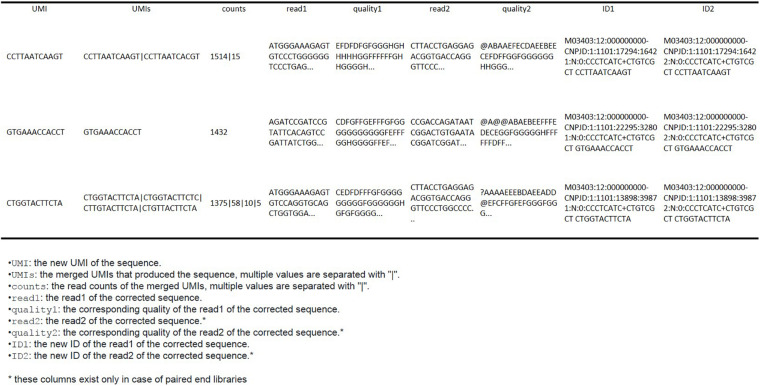
Example of the output csv table with all relevant information of the fastq files.

The framework also produces a “*summary_table.csv*” including all the information of the output fastq files, as well as extra information that can help the user to return from the output sequences to their corresponding input sequences. It is organized in a table (Figure 3), in which each row is an output sequence.

## Results

### Implementation

The proposed framework for the deduplication and error correction of UMI libraries was evaluated on two different kinds of library preparation steps. The first dataset is a UMI-tagged paired-end library generated as described in Girardot et al. (2016). In this case, the UMIs are 12nt long and are at the very beginning of R1. The second dataset is also UMI-tagged paired-end library and contains two different samples generated by the approach of Zilionis et al. Orabi et al. (2019). Here, the UMIs are 10 nt long and are at the very beginning of R1 and R2. The implementation was performed based on the available datasets and on different use cases: (i) single-end libraries: UMI on R1, (ii) paired-end libraries: UMI on R1, and (iii) paired-end libraries: UMI on R1 and R2. The use case (ii) contains the first dataset selected for the following section avoiding repetitions to be mentioned in all the use cases with the related datasets.

### Complexity

Regarding the theoretical complexity, it can be roughly calculated in the following way. First, we define the following constants:

• *m*: number of sequences
• *n*: length of sequences
• *k*: length of UMIs

We proceed to an estimation of the theoretical complexity, by splitting the workflow of the toolkit into its most basic parts. Just a reminder that the workflow is analytically presented both on paper and in the wiki method page of the GitHub repository as well.

(1) Data cleaning: *O*(*m*⋅*k*)
(2) Read correction: *O*(*k*⋅*m*^2^)+*O*(*m*⋅*n*), because

(a) Identifying the same UMIs: *O*(*k*⋅*m*^2^)
(b) Read correction: *O*(*m*⋅*n*)

(3) UMI merging: *O*(*k*⋅*m*^2^)+*O*(*n*⋅*m*^2^)

(a) Hamming distance between first UMI and the rest of them: *O*(*k*⋅*m*)
(b) Calculation of Hamming distances between sequences: *O*(*m*⋅*n*)
(c) For each UMI: *O*(*m*)×{*sumofpreviouscomplexities*}

(4) Final read correction: *O*(*k*⋅*m*^2^)+*O*(*m*⋅*n*)

So, an overall estimation of the theoretical complexity is

$$O\,\left[\left(k+n\right)\cdot m^{2}+\left(k+n\right)\cdot m\right]$$

It is important to mention here that this is a very rough estimation of the complexity, but it is definitely an upper bound. For example, the complexity of read correction part consists of two terms: *O*(*k*⋅*m*^2^) and *O*(*m*⋅*n*). The first term is associated with finding identical UMIs and the second one in applying the reading correction process in the sequences. However, there is definitely a trade-off between the two terms. At an extreme scenario where all UMIs are identical, the second term reaches its highest value *O*(*m*⋅*n*), whereas the first one degenerates to *O*(*m*⋅*k*). The latter scenario could be also considered as the best-case scenario, in which the computation time reaches an Ω[(*n* + *k*)⋅*m*] complexity. In contrast, at a scenario where all UMIs are unique, the first term reaches its highest value *O*(*k*⋅*m*^2^), but there is no read correction process, so the second term is zeroed. Same holds, more or less, at the UMI merging part. In practice, the computation time seems to grow almost linearly with respect to sample size *m*.

We generated 20 artificial datasets by randomly sampling different numbers of rows from the original dataset. The datasets consisted of 1,000 to 1,000,000 reads and were used as input to UMIc, selecting case 1 as a workflow (paired-end libraries and UMI in Read1). Below we present a table containing the computational time of UMIc for each one of the artificial datasets and the number of Hamming distances (Table 2). All experiments were performed on a 24-core Unix cluster with 220 GB RAM.

**TABLE 2:** A table containing the computational time of UMIc for each one of the artificial datasets.

Dataset	Number of reads	Execution time in seconds	Execution time in minutes	Number of Hamming distances calculated
1	1,000	337.8267	5.63	2,090
2	2,000	394.6571	6.58	3,588
3	5,000	533.7972	8.9	7,533
4	10,000	631.97	10.54	12,633
5	20,000	726.3552	12.106	18,484
6	50,000	987.8132	16.4636	32,088
7	100,000	1,815.77	30.263	53,374
8	150,000	2,308.1	38.47	77,602
9	200,000	3,019.3	50.32	103,124
10	250,000	3,614.9982	60.25	126,852
11	300,000	4,116.79	68.62	160,376
12	350,000	3,756.78	62.613	193,272
13	400,000	2,912.381	48.5396	224,337
14	450,000	2,924.56	48.74	245,492
15	500,000	4,743.065	79.051	281,483
16	600,000	7,120.2394	118.67	358,086
17	700,000	4,144.6042	69.076	414,266
18	800,000	5,476.5872	91.2764	456,569
19	900,000	7,245.7027	120.7617	465,044
20	1,000,000	7,967.4016	132.78	488,868

*Apart from the computational time (in seconds and minutes), we also present the number of Hamming distances that are calculated for each run.*

**FIGURE 4:**
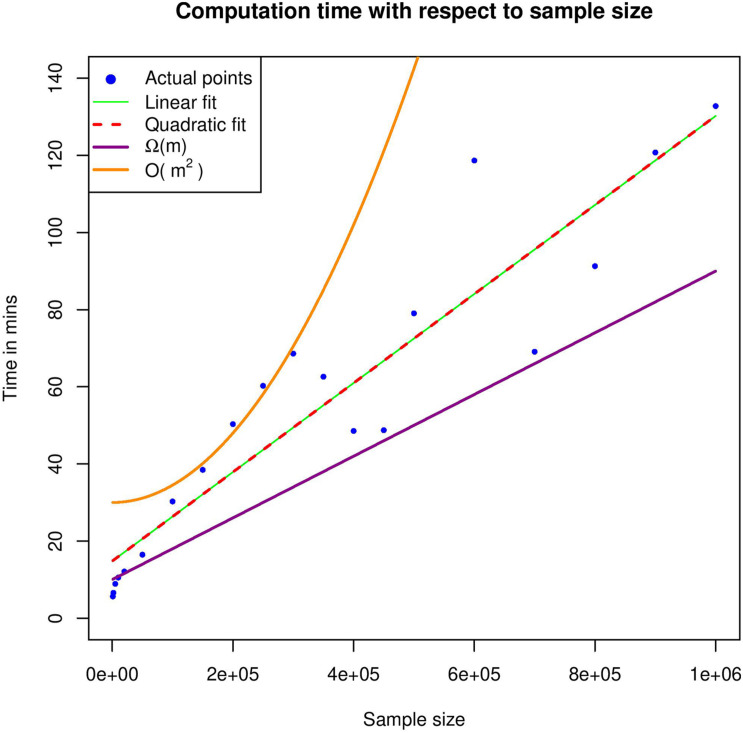
Execution time with respect to different sample sizes. In the graph, the actual time points were fit to a linear and a quadratic function. Moreover, an upper and a lower bound of the estimated complexity is also included.

Figure 4 shows the computational time required for various datasets and therefore provides an estimate of the underlying complexity. In order to identify the best-fitting function, we used both linear and quadratic estimates; however, quadratic fit seems to be a degenerated version of the linear one, which further enhances our argument that our theoretical analysis corresponds to the worst-case scenario. In practice, computation time seems to grow linearly with respect to sample size.

### Case Report

**FIGURE 5:**
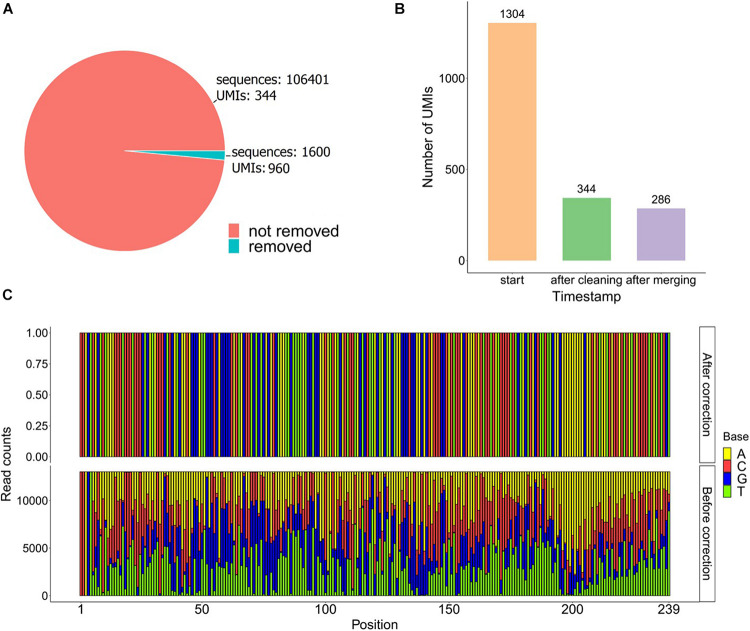
**(A)** Pie chart of the sequences before and after the data-cleaning step. **(B)** Bar plot of the UMIs found on the raw fastq file (start) and the UMIs remaining after the cleaning and the merging steps. **(C)** Frequency bar plot of the bases from 13,020 reads in each position using the UMI GTAAAACGACGG after and before correction.

**FIGURE 6:**
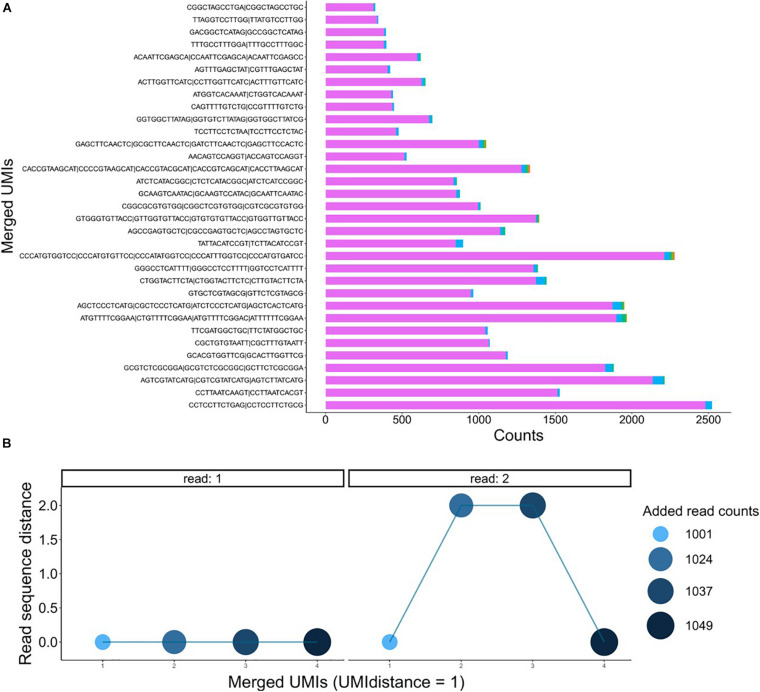
**(A)** Bar plots showing the number of reads on the merged UMIs. **(B)** Dot plots of a group of four UMIs on the *x*-axis and the distance of the reads on the *y*-axis on R1 and R2. The density and the size of the dots represent the number of reads after merging.

The used dataset contains 108,001 reads, and we found 1,304 UMIs after extracting the first 12 bp of the R1 fastq file from a UMI-tagged library. Applying the data-cleaning step using the countsCutoff resulted in 106,401 reads with 344 related UMIs (Figure 5A). After the merging of the UMIs, we ended up with 286 unique DNA molecules (criteria: UMI distance = 1, sequence distance = 3, min counts cutoff = 6) (Figure 5B). In other words, the input fastq file contains 108,001 reads, and the output fastq files include the 286 UMIs resulting from the UMIc workflow. The process collapses the reads derived from the same UMI and contributes to the creation of the consensus sequence. An example of a UMI with the bases before and after correction is displayed in Figure 5C. The UMIs were merged and corrected based on our approach, and 33 UMIs that showed merging with other UMIs were displayed in Figure 6A. Particularly, we examined the merging and correction steps of a random read (M03403:12:000000000-CNPJD:1:1101:15600:2169 1:N:0:CCCTCATC+CTGTCGCT). The UMI of the read was GAGCTTCAACTC, and we found 1,001 reads with the same UMI. Then, after scanning the other reads that met the criteria of distance, we found three more groups of reads with the UMIs G**C**GCTTCAACTC, GA**T**CTTCAACTC, GAGCTTC**C**ACTC and number of reads 23, 13, and 12, respectively (Figure 6B). The R1 showed no distance between the actual reads, and the R2 showed a distance range from 0 to 2 bases.

### Comparison to Relevant Approaches

We used UMI-tools (Smith et al., 2017), a software toolbox for dealing with UMIs and single-cell RNA-Seq cell barcodes and pRESTO (Vander Heiden et al., 2014), a toolkit for processing raw reads from high-throughput sequencing of B-cell and T-cell repertoires including features for UMIs, in order to compare its functionalities with UMIc. The main differences are listed in Table 3.

**TABLE 3:** Comparison of functionalities offered by UMIc, UMI-tools, and pRESTO.

Feature	UMIc	UMI-tools	pRESTO
Language	R	Python	Python
Input	fastq	bam	fastq
Align free	Yes	Not supported	Yes
Sequence length	Supports only same sequence length	Supports different sequence lengths	Supports different sequence lengths
Extract UMI	Based on the number of nucleotides on 5′	Based on the pattern of barcodes on 5′ and 3′	Based on the number of nucleotides on 5′
Paired data	Yes	Yes	Yes
UMI on R1 or R1 and R2	Yes	Yes	Yes
UMI correction	Through UMI and reads distance	Offers five methods (three of them network based, which use UMI distance and read counts) and one of them cutoff 1% of mean (number of reads/UMI)	In case of significant nucleotide diversity within UMI groups, divides the groups in subclusters
Data cleaning	Specification of min number of reads/UMI group	UMI quality filtering for a specified Phred score threshold	UMI quality filtering for a specified Phred score threshold and offers removal of highly variable UMI read groups
Deduplication	Creation of consensus sequence, using per base frequency and Phred scores	Selection of representative read, based on mapping coordinates and quality	Creation of consensus sequence, using per base Phred scores (optionally, a frequency and quality threshold that will assign an N to the position)
Output	fastq	bam	fastq

A clear advantage of UMIc is that, by omitting the alignment step to a reference genome, it allows us to analyze data from hypervariable regions, such as the B- and T-cell receptor sequences (BCR and TCR sequencing). Traditional alignment/mapping tools such as bowtie that are used on UMI-tools are unable to analyze this kind of data, given that none of the reference genomes (hg19 or hg38) include information of the V(D)J construction. In this perspective, UMIc’s alignment-free approach has the advantage of excluding the UMIs during analysis, and deduplicating the corresponding sequences, therefore building a unique consensus without the need of chromosomal location information, which is very complex in the cases of BCR and TCR sequencing. Indeed, testing and validation of UMIc were performed using BCR sequencing data; it is important to highlight that UMI-tools are not able to effectively process them. However, and in order to provide an effective comparison between UMI-tools and UMIc, we used the test dataset provided by UMI-tools and our own validation dataset independently, after ensuring that their respective characteristics are comparable. Specifically, UMI-tools provide a test dataset that contains ∼1 million reads after mapping, with the read length ranging from 22 to 76 bps. In order to perform an appropriate comparison, for UMIc, we also used single-end raw reads (∼1 million), after manually trimming them to the maximum length of the corresponding reads from UMI-tools (i.e., 76 bp).

For UMI-tools, the UMI extraction required 158 s (∼2.5 min) to complete, the alignment step to the reference genome was completed in ∼20 min, and the deduplication step was performed in 69 s (∼1 min). Overall, the UMI-tools workflow needed ∼24 min to complete. UMIc was able to complete the entire process in 990.175 s (∼16.5 min). Regarding the memory requirements of the UMIc workflow, after data cleaning, the overall memory used was ∼890 MByte. After building the first consensus across all reads, the memory footprint increased to ∼932 MByte and finally decreasing to ∼878 MBytes after UMI merging. All experiments for both tools were performed on the same UNIX-based environment with 220 GB RAM available and using only a single thread.

**FIGURE 7:**
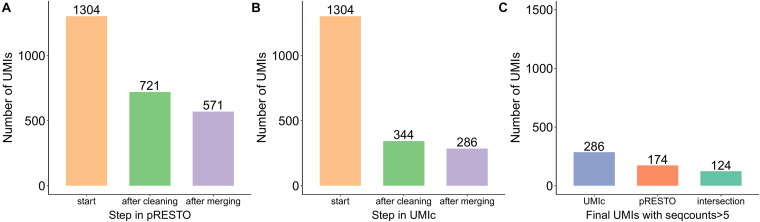
Bar plots showing the number of UMIs processed at each step by **(A)** pRESTO and **(B)** UMIc as well as **(C)** the common UMIs of the two tools.

As an additional comparison, we used pRESTO, which is a toolkit for processing raw reads from high-throughput sequencing of B- and T-cell repertoires. However, pRESTO requires a file containing primer sequences in order to properly identify the UMIs. As our tool was tested on two different data types produced by different protocols (Stahlberg et al. and Zilionis et al.), only the former one contained primer sequences. The latter does not contain any primers (as the underlying protocol does not require primer sequencing) and therefore could not be analyzed by the pRESTO tool. Ultimately, the dataset that was used to evaluate both pRESTO and UMIc contains 1,304 UMIs; 571 distinct UMIs were identified by pRESTO and 286 by UMIc. However, UMIc also takes into consideration the number of reads present in each UMI. By applying the same filtering on the results generated by pRESTO, we ended up with 174 UMIs remaining from pRESTO (Figure 7A), of which 124 UMIs were common to the 286 UMIs produced by UMIc (Figure 7B), clearly highlighting the discovery sensitivity of our approach (Figure 7C). To summarize, our approach (i) does not require a primer sequencing file and is applicable on (ii) all the NGS experiments (RNA-seq, DNA-seq, etc.) not only on BCR-seq data and (iii) the different library preparation protocols.

## Discussion

Unique molecular identifiers can be used to identify PCR duplicates from the amplification steps on NGS experiments (Kivioja et al., 2011; Islam et al., 2014). By adding a random UMI in each read, it is possible to exclude duplicates based on the unique UMIs. UMIs are introduced in many NGS experiments such as RNA-seq, single-cell RNA-seq (scRNA-seq) (Zilionis et al., 2017; Srivastava et al., 2019), BCR repertoire sequencing (Egorov et al., 2015), etc. Also, it is worth noting that the UMI approach is suitable for NGS-based measurable residual disease detection, which allows the determination of individual risk in acute myeloid leukemia patients based on mutational clearance after treatment (Yoest et al., 2020) and the IG/TR rearrangements in acute lymphoblastic leukemia (Bruggemann et al., 2019). However, and despite the increase in bioinformatics tools for UMI analysis, there exists no single approach to efficiently remove UMIs and correct reads accordingly.

In this work, we propose a novel tool, namely, UMIc, which provides a complete framework to analyze UMI-tagged libraries. Our approach is easily applicable to any type of fastq files with the prerequisite of the existence of UMIs at the beginning of the reads. The UMIc implementation supports a fast execution for generating (i) the corrected fastq files and (ii) a table in csv format including additional information of the output fastq files.

In relation to other existing tools, UMIc is a preprocessing step skipping the alignment step and deduplicates and corrects reads directly based on the DNA sequence. By omitting the alignment, our method is faster on larger datasets. The output contains corrected fastq files in which each read emerges from the deduplication of the UMIs during the generation of a consensus sequence. The criterion of the consensus sequence takes into account both the frequency and the mean Phred quality of nucleotides.

A number of tools have been developed that contain a fixed workflow including the sequence assembly, such as migec (Shugay et al., 2014) and pRESTO (Vander Heiden et al., 2014), tailored to the analysis of BCR and TCR repertoire sequencing. Their design has been mainly driven by the specificity of the data, which cannot be readily analyzed with traditional mappers such as bwa, bowtie2, and others. The UMIc approach of omitting the mapping on a reference genome addresses this challenge by supporting its use as a preprocessing step. This means that the user can, after the application of UMIc, continue the analysis on their pipeline of preference such as IMGT (Alamyar et al., 2012), HISAT2 (Kim et al., 2019), or bwa (Li and Durbin, 2009), using the fastq files produced as output from the proposed framework. To achieve this, UMIc takes into account the distances of sequences separating the UMI and the actual read. The UMI meeting the criteria of distance with the other UMIs (e.g., 1 bp) is examined for the distances between the remaining sequences. Moreover, the duplicated sequences will result in a short distance between the actual reads due to sequencing errors.

Overall, UMIc provides a complete framework to analyze UMI-tagged libraries. These libraries need to go through a demultiplexing and error-correcting process, based on the unique UMIs that serve as monitors of the original module (e.g., a DNA fragment). We see UMIc as a broad-use tool, similarly to other trimming and preprocessing tools, such as Trim Galore! which produces correcting and adaptor-free reads. In the same overall philosophy, our approach serves as a preprocessing step of fastq files for deduplication of reads and also an error correction step, toward building consensus sequences from each UMI. The significant novelty of UMIc is that, by skipping the alignment step, it enhances the downstream analysis and therefore can be directly added to several NGS workflows as a preprocessing step. This allows the user to be able to use any tools based on preference and the respective NGS experiments; for example, if the input fastq files are DNA-seq, the user can perform the alignment process using bwa directly on the output of UMIc. This approach offers a wide application of the UMIc tool completely independently of the overall selected computational pipeline (RNA-seq, DNA-seq, and BCR-seq).

UMIc has been implemented as an open-source R package, in accordance with the FAIR principles (Findable, Accessible, Interoperable, Reusable) for research software (Lamprecht et al., 2020). The tool is freely available on GitHub^1^ under an MIT license, including detailed documentation of installation and implementation, highlighting the reproducibility of the source code.

Overall, there is a growing need for appropriate tools for error correction on NGS experiments. The use of UMIs is promising, yet because of their recent implementation, the downstream analysis of these kinds of NGS data is still an ongoing process. Our method gives a new perspective toward analyzing UMIs by offering a short execution time in R language, which provides the opportunity of generating corrected fastq files from the initial raw data.

## Data Availability Statement

The original contributions presented in the study are included in the article/supplementary material and through the GitHub repository (https://github.com/BiodataAnalysisGroup/UMIc), further inquiries can be directed to the corresponding author.

## Author Contributions

MT designed the study and wrote the manuscript. MCM, AT, and NP developed the tool, analyzed the data, and wrote the manuscript. MK and TH provided the data and reviewed the submitted version. IK reviewed the manuscript. AC supervised the study and reviewed the submitted version. FP designed and supervised the study, and reviewed the manuscript. All authors contributed to the article and approved the submitted version.

## Conflict of Interest

The authors declare that the research was conducted in the absence of any commercial or financial relationships that could be construed as a potential conflict of interest.

## Footnotes

1 https://github.com/BiodataAnalysisGroup/UMIc
